# The effect of a combined rehabilitation program on the temporomandibular joint in systemic sclerosis evaluated by ultrasound exam

**DOI:** 10.1007/s40477-023-00839-8

**Published:** 2023-12-14

**Authors:** Daniela Melchiorre, M. Passalacqua, M. Maresca, G. Landi, M. A. Bagni, K. El Aoufy, M. Baccini, M. Matucci-Cerinic, S. Maddali Bongi

**Affiliations:** 1 Division of Rheumatology, Department of Experimental and Clinical Medicine, University of Florence, Viale Largo Brambilla 3, 50134 Florence, Italy; 2 AMURR, Associazione Multidisciplinare Riabilitazione Reumatologica, Florence, Italy; 3 UNIRAR, Ospedale San Raffaele, Milan, Italy

**Keywords:** Temporomandibular joint, Systemic sclerosis, Ultrasound examination, Rehabilitation

## Abstract

**Purpose:**

Temporomandibular joint (TMJ) involvement is frequent in Systemic Sclerosis (SSc). Dysfunction and X-ray changes of TMJ were described only in few observational studies. Treatment as well has been seldom considered. Aim of the present study was to evaluate the effects on TMJ of two specifically designed physiotherapy protocols.

**Methods:**

The study group included 26 SSc outpatients (22 females and 4 males with mean age ± SD 59.08 ± 10.31 years). Thirteen patients were randomly assigned to a treatment (protocol 1) including home exercises for TMJ and thirteen to a treatment (protocol 2) including home exercises and a combined procedure. The rehabilitation effects on the TMJ were evaluated by ultrasound examination (UE) in static and dynamic phases. UE was performed in all patients before and at the end of the treatment and after a follow up (8 weeks).

**Results:**

Both rehabilitation protocols induced a significant improvement (protocol 1: p < 0.01 and protocol 2: p < 0.005) of mouth opening with a long-lasting effect. Protocol 2 was more effective than protocol 1. A significant increase of bilateral condyle-head temporal bone distance was detected by UE at the end of both treatments. It was maintained at follow-up in patients treated with Protocol 2.

**Conclusions:**

The present investigation shows that a rehabilitation program characterized by home exercises with a combined procedure is useful to recover the function of TMJ. The data also show that UE is helpful in the evaluation of TMJ in SSc and in the assessment of the efficacy of the rehabilitation programs.

## Introduction

Systemic sclerosis (SSc) is a complex autoimmune disorder involving the skin, the musculoskeletal system and internal organs and characterized by vascular abnormalities, fibrosis and atrophy of the skin and subcutaneous tissue [1]. The modification of the facial tissues leads to the typical "facies sclerodermica" with ipo- or amimia and microstomia and microcheilia [2]. Consequently, the TMJ involvement is frequent [3–6]. Smirani et al. [7] reported in SSc patients the frequency of TMJ symptoms from 92.5% to 94.8%, while condyles erosion was observed from 2.4% to 20% of patients. In a recent review [8] is reported that in SSc patients the percentage related to TMJ symptoms and impaired jaw functionally was between 20 and 93%. oreover, has also been described that 81.5% of patients showed by MRI a disk displacement with reduction and 67% of patients degenerative bone changes [8]. Typical TMJ X-ray findings are mainly bone resorption of the jaw angle and of the condyles, seldom with fractures [9–12]. It is important to note that dysfunction and X-ray TMJ changes were described only in a few observational studies [5, 6]. Moreover, only few studies proposed treatments [13–16]. Even if surgery (bilateral commissurotomy) is sometimes required for the treatment of severe microstomia [9, 10], good results may be obtained by rehabilitation [13–17]. As Kumar et al. [18] recently observed, ultrasound examination (UE) is an imaging modality which can accurately show changes of the hard and soft tissues in TMJ. With UE it is possible to visualize the static and dynamic relationship of joint structures with open and closed mouth, as also demonstrated by Galletti et al. [19]. Previously, we have studied the TMJ with the UE to evaluate the inflammatory process involving TMJ in rheumatic diseases [20–23]. In other disorders, UE is used in the evaluation of the efficacy of rehabilitation programs [24].

Aim of the present study was to verify the effects of two specifically designed physiotherapy protocols on the TMJ in SSc patients.

## Methods

Twenty-six SSc patients (22 females and 4 males with mean age ± SD 59.08 ± 10.31 years and disease duration ± SD 13.65 ± 5.71 years) were enrolled from the outpatient clinic of the Rheumatology Unit, Department of Experimental and Clinical Medicine, of the University of Florence.

Inclusion criteria were: SSc classified according to ACR/EULAR criteria [25] and at least one of the following characteristics: (1) Measurement of mouth opening ≤ 40 mm; (2) MHISS [Mouth Handicap in Systemic Sclerosis stairs) Score ≥ 8 [26]; 3) Helkimo clinical disfunctional index > 1 [27].

The study was approved by the local ethics committee (711/12) and patients signed a written informed consent form.

At enrollment, patients were evaluated for clinical and demographic data (sex, years from diagnosis and disease subset [limited or diffuse cutaneous subset SSc (lSSc or dSSc) [28], organ (skin, lung, heart, gastrointestinal, renal) involvement and antibody pattern, according to international guidelines [29].

After the enrollment, patients were randomly assigned to protocol 1 or protocol 2. Randomization was performed using a numerical sequence prepared by a person not connected to the study, which provided sequentially numbered and sealed envelopes. The result of the randomization remained unknown until the participant did not decide to accept or decline participation in the study. Thirteen patients were assigned to Protocol 1 (group 1) and thirteen patients to Protocol 2 (group 2).

**Protocol 1:** Home exercises (20 min/day, 3 times/week) for TMJ, mimic, masticatory and cervical spine muscles.

**Protocol 2:** Home exercises (as in Protocol 1) and a combined procedure (1/week; 45 min/session) including connective tissue massage [30] of face and neck, Kabat technique [31] applied to mimic muscles, manual techniques (intra- and extra-oral TMJ manipulation, stretching and mobilization of the cranio-cervical district).

All patients continued their drug treatment and did not begin any physiotherapy treatment unrelated to the study.

Each protocol had a total duration of 20 weeks (5 months): 12 weeks (3 months) of treatment and 8 weeks (2 months) of follow-up. The patients were assessed at enrollment (T0), after 12 weeks of treatment (T1) and after 8 weeks of follow-up (T2).

## Assessment

1. **Mouth opening**: the maximum mouth opening was evaluated as a mean (in cm) of 2 measurements of the distance between central incisors (from the lower edge of the upper incisors to the upper edge of the lower incisors) adding the amount of vertical incisor overbite. A Thera Bite device was used [32];
2. **Clinical evaluation of TMJ:** Evaluation of joint play, joint sounds (clicks or crackling) and TMJ pain induced by palpation or TMJ movement (opening, closing, laterality, protrusion);
3. **MHISS (Mouth Handicap in Systemic Sclerosis scale):** mouth handicap in SSc is evaluated by 12 items (ranging score of each item = 0–4); the total score (range 0–48) is divided into three partial scores concerning: (1) the disability related to the reduced mouth opening (5 items; ranging score 0–20), (2) the handicap correlated to Sicca syndrome (5 items; ranging score 0–20), (3) aesthetic problems (2 items; ranging score 0–8) [26];
4. **Helkimo Index** [27]: it is a score ranging from 0 to 25 (0 = absence of clinical symptoms; 1–4 = minor dysfunction; 5–9 = moderate dysfunction; 10 – 25 = severe dysfunction);

The TMJ involvement was evaluated by x-ray (Orthopantomography) only at T0 and by UE at T0, T1 and T2: UE examination, in static and dynamic phases with a linear probe 8–16 MHz (My LAB 70 X Vision, ESAOTE S.p.A., Milan, Italy), was performed in all patients by the same sonographer (DM) blinded to the protocol of each patient. The probe was placed along the axis of the mandibular branch for the static evaluation. The study of TMJ in the dynamic evaluation was performed with the probe maintained along the axis of the mandibular branch to follow the condyle excursion and to detect the disc position that was also studied placing the probe on the same plane of zygomatic arch [19]. The distance of the condyle-head temporal bone was detected at maximum mouth opening with the probe head placed on the temporal articular tubercle. The study of masseter muscle was performed with the probe placed between zygomatic arch and inferior mandibular edge. The following TMJ characteristics were evaluated in each joint: (1) joint space; (2) presence of joint effusion; (3) condylar profile, osteophytis and/or erosions; (4) position of the articular disc at open and closed mouth; (5) power Doppler activity; (6) distance condyle-head temporal bone at the point of maximum mouth opening (mean of two measurement); (7) measurement of masseter muscle thickness at rest; (8) measurement of masseter muscle thickness during forced clench [20–23].

### Statistical analysis

The unpaired t test was used to compare data between the two groups at different times of the study. Data analysis was performed using the statistical program SPSS for Windows.

## Results

Mean values ± SD of mouth opening, MHISS and Helkimo Index at T0, T1 and T2 in patients treated with Protocol 1 and protocol 2 are reported in Table 1a, b. *P* values obtained in the comparison of data are also reported.

**Table 1:** Clinical findings at T0 (before treatment), T1 (end of treatment) and T2 (follow up after 8 weeks) in patients treated with protocol 1 (a) and protocol 2 (b)

(a)								
Protocol 1	T0		T1		T2		T1/T0	T2/T0
	Mean	± SD	Mean	± SD	Mean	± SD	*p* value	*p* value
Mouth opening mm	39.10	± 4.58	44.90	± 5.43	44.60	± 5.04	< 0.01	< 0.01
MHISS_total	27.20	± 9.66	21.90	± 11.69	21.40	± 10.78	ns	ns
MHISS_mouth opening	12.50	± 5.15	10.10	± 5.24	9.80	± 4.89	ns	ns
MHISS_sicca syndrome	10.00	± 5.42	8.20	± 5.53	8.44	± 4.98	ns	ns
MHISS_aesthetic problems	4.70	± 2.45	3.60	± 2.88	4.00	± 2.87	ns	ns
Helkimo Index	13.00	± 5.62	8.60	± 4.62	10.00	± 4.16	< 0.05	ns

(b)								
Protocol 2	T0		T1		T2		T1/T0	T2/T0
	Mean	± SD	Mean	± SD	Mean	± SD	*p* value	*p* value
Mouth opening mm	46.00	± 4.12	51.00	± 3.19	51.60	± 3.50	< 0.005	< 0.001
MHISS_total	15.91	± 12.69	13.27	± 11.76	14.20	± 11.06	ns	ns
MHISS_mouth opening	6.55	± 6.8	5.36	± 5.54	6.80	± 5.07	ns	ns
MHISS_sicca syndrome	7.55	± 6.61	6.36	± 6.05	5.70	± 5.72	ns	ns
MHISS_aesthetic problems	1.73	± 2.41	1.55	± 1.44	1.70	± 1.57	ns	ns
Helkimo Index	8.09	± 3.99	5.55	± 4.08	7.50	± 5.19	ns	ns

### Sonographic findings

1. Patients treated with protocol 1: the sonographic measurable findings of the right TMJ (mean ± SD) are the following: joint space: 1.82 ± 0.51 (T0), 1.82 ± 0.31 (T1), 1.63 ± 0.42 (T2); distance condyle-head temporal bone at the point of maximum mouth opening: 12.66 ± 1.75 (T0), 13.41 ± 1.91 (T1), 14.15 ± 2.09 (T2); masseter muscle thickness at rest 8.02 ± 1.47 (T0), 8.48 ± 1.81 (T1), 8.05 ± 0.71 (T2) and during forced clench 11.68 ± 2.29 (T0), 11.39 ± 2.32 (T1), 11.00 ± 1.70 (T2). The findings of the left TMJ (mean ± SD) in the group 2 are the following: joint space: 2.17 ± 0.78 (T0), 2.25 ± 0.92 (T1), 1.82 ± 0.44 (T2); distance condyle-head temporal bone at the point of maximum mouth opening: 12.79 ± 1.40 (T0), 14.15 ± 2.04 (T1), 14.33 ± 1.74 (T2); masseter muscle thickness at rest 8.23 ± 1.30 (T0), 8.36 ± 1.62 (T1), 8.35 ± 1.62 (T2) and during forced clench 11.64 ± 2.47 (T0), 11.22 ± 2.07 (T1), 11.20 ± 2.04 (T2). Bilateral joint effusion was detected in 8/13 (61.5%) pts. An altered condylar profile was present on the right side in 10/13 (76.9%) pts and on the left side in 9/13 (69.2%) pts. In 2/13 (15.3%) pts was present osteophytes on the left and in 1/13 (7.6%) on the right. In 1/13 (7.6%) pts an erosion was detect on the right. The articular disc appeared to be displaced anteriorly on the right in 1/13 pts (7.6%). In no patient there were signs of activity at power Doppler.
2. Patients treated with protocol 2: the sonographic measurable findings of the right TMJ (mean ± SD) are the following: joint space: 1.79 ± 0.69 (T0), 1.88 ± 0.49 (T1), 1.83 ± 0.52 (T2); distance condyle-head temporal bone at the point of maximum mouth opening: 12.00 ± 2.35 (T0), 14.25 ± 1.95 (T1), 14.51 ± 2.45 (T2); masseter muscle thickness at rest 7.85 ± 1.47 (T0), 7.75 ± 0.8 (T1), 7.75 ± 0.8 (T2) and during forced clench 10.95 ± 1.73 (T0), 11.40 ± 1.73 (T1), 11.40 ± 1.73 (T2). The findings of the left TMJ (mean ± SD) in the group 1 are the following: joint space: 1.42 ± 0.29 (T0), 1.46 ± 0.30 (T1), 1.56 ± 0.23 (T2); distance condyle-head temporal bone at the point of maximum mouth opening: 12.20 ± 1.68 (T0), 14.35 ± 1.35 (T1), 15.16 ± 1.47 (T2); masseter muscle thickness at rest 8.34 ± 1.48 (T0), 7.86 ± 0.90 (T1), 8.75 ± 0.8 (T2) and during forced clench 11.26 ± 1.99 (T0), 11.41 ± 1.80 (T1), 11.40 ± 1.83 (T2). Bilateral joint effusion was detected in 3/13 pts (23%). An altered condylar profile was present on the right side in 9/13 (69.2%) pts and on the left side in 5/13 pts (38.4%). In 2/13 (15.3%) pts osteophytes were present on the left and in 1/13 (7.6%) on the right. In 1/13 (7.6%) pts an erosion was detect on the left. The articular disc appeared to be displaced anteriorly in 1/13 (7.6%) pts on the right and on the left in 2/13 (15.3%) pts. In no patient there were signs of activity at power Doppler.

**Fig. 1:**
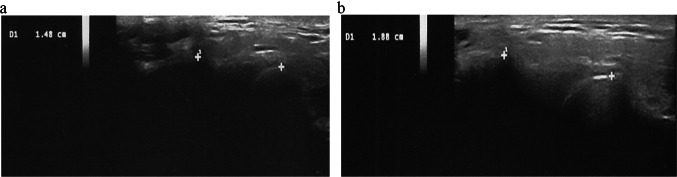
**a** Ultrasound image of temporomandibular joint in a SSc patient treated with protocol 2: distance condyle-head temporal bone at the point of maximum mouth opening before the treatment. The calipers are placed: in the middle point of the convexity of the condyle and on the temporal articular tubercle. **b** Ultrasound image of temporomandibular joint in the same SSc patient after treatment with protocol 2

In the patients treated with protocol 1 the distance condyle-head temporal bone was increased on both sites at T1 and at T2, but the differences were not significant (Table 2). In the patients treated with protocol 2 the distance condyle-head temporal bone was increased (Fig. 1); on the right at T1 (p < 0.05) and at T2 (< 0.05), on the left at T1 (< 0.005) and at T2 (< 0.001) (Tab 2).

**Table 2:** Condyle-temporal bone distance and masseter muscle thickness at rest and during forced clench at T0 (before treatment), T1 (end of treatment) and T2 (follow up after 8 weeks) in patients treated with protocol 1 and protocol 2

c	T0		T1		T2		T1/T0	T2/T0
	Mean	± SD	Mean	± SD	Mean	± SD	*p* value	*p* value
Right condyle-temporal bone distance	12.66	± 1.75	13.41	± 1.91	14.15	± 2.09	ns	ns
Left condyle-temporal bone distance	12.79	± 1.40	14.15	± 2.04	14.33	± 1.74	ns	ns
Right masseter muscle thickness at rest	8.02	± 1.47	8,48	± 1.81	8.05	± 0.71	ns	ns
Right masseter muscle thickness during forced clench	11.68	± 2.29	11.39	± 2.32	11.00	± 1.70	ns	ns
Left masseter muscle thickness at rest	8.23	± 1.30	8.36	± 1.62	8.35	± 1.62	ns	ns
Left masseter muscle thickness during forced clench	11.64	± 2.47	11.22	± 2.07	11.20	± 2.04	ns	ns
*Protocol 2*								
Right condyle-temporal bone distance	12.00	± 2.35	14.25	± 1.95	14.51	± 2.45	< 0.05	< 0.05
Left condyle-temporal bone distance	12.20	± 1.68	14.35	± 1.35	15.16	± 1.47	< 0,005	< 0.001
Right masseter muscle thickness at rest	7.85	± 1.47	7,75	± 0.8	7.75	± 0.8	ns	ns
Right masseter muscle thickness during forced clench	10.95	± 1.73	11.40	± 1.73	11.40	± 1.73	ns	ns
Left masseter muscle thickness at rest	8.34	± 1.48	7.86	± 0.90	8.75	± 0.8	ns	ns
Left masseter muscle thickness during forced clench	11.26	± 1.99	11.41	± 1.80	11.40	± 1.83	ns	ns

## Discussion

In SSc, it is still an open issue whether the TMJ arthropathy is due to TMJ bone changes or to perioral tissue fibrosis [4–8]. In different studies carried out since 1984 [13] exercise programs, when applied, were useful to improve the mouth opening of SSc patients [14]. Maddali et al. [17] demonstrated that facial and TMJ rehabilitation is useful for improving mouth movement and reducing pain in SSc patients. Add effects of two rehabilitation programs were compared showing an improvement of some clinical and clinimetric variables with a long-lasting effect. Moreover, the effects of the physiotherapist prescribing and personalizing exercise may induce better results.

The data of our study are in agreement with those of Kumar et al. [18] concerning the fact that UE is useful for the detection of the pathologic modifications of the TMJ in SSc patients. An altered condylar profile, disc displacement and osteophytes were present bilaterally while erosions were detected in 7.6% patients only. These results are consistent with data reported in other studies [4, 7, 8, 33]. Moreover, in the present investigation bilateral joint effusion was frequently detected but no signs of activity were found at power Doppler. As previously reported, this latter finding is an important feature of TMJ involvement and can be easily observed by UE [19–23].

In the present study two different rehabilitation protocols were used in SSc patients with TMJ involvement. Protocol 1 included only home exercises, Protocol 2 included home exercises and connective tissue massage, Kabat technique and manual techniques performed by a physiotherapist. Both rehabilitation protocols induced an improvement of mouth opening but a long-lasting effect was maintained only by Protocol 2. It is noteworthy that UE found a significant increase of bilateral condyle-head temporal bone distance at the end of treatment and at the follow-up only in patients treated with protocol 2.

Our data confirm that UE is an important tool in monitoring the rehabilitative follow-up in SSc patients. It is important to note that in this population bone damage findings were observed more frequently than disc displacement and signs of inflammatory TMJ involvement, as observed in other rheumatic diseases [20–23]. Only a few investigations were previously devoted to the study of TMJ modifications and oral impairment in SSc patients [4–7]. It is also important to consider that the increase of mouth opening, obtained with rehabilitation, allows an improvement of oral tissue lesions and related symptoms. Therefore, TMJ-UE should be recommended in every patient with SSc to detect pathologic changes in early phase and to avoid the severe consequences of the reduction of mouth opening and the decay of oral health. We should also consider that UE has proven to be very useful in both diagnosis and follow-up in many other fields of medicine as well [34–36].

## Data Availability

The data are the results reported in the article and are available.
